# Neuroprotectin D1 upregulates Iduna expression and provides protection in cellular uncompensated oxidative stress and in experimental ischemic stroke

**DOI:** 10.1038/cdd.2017.55

**Published:** 2017-04-21

**Authors:** Ludmila Belayev, Pranab K Mukherjee, Veronica Balaszczuk, Jorgelina M Calandria, Andre Obenaus, Larissa Khoutorova, Sung-Ha Hong, Nicolas G Bazan

**Affiliations:** 1 Neuroscience Center of Excellence, School of Medicine, Louisiana State University Health Sciences Center, New Orleans, LA, USA; 2 Department of Pediatrics, Loma Linda University, Loma Linda, CA, USA

## Abstract

Ring finger protein 146 (Iduna) facilitates DNA repair and protects against cell death induced by NMDA receptor-mediated glutamate excitotoxicity or by cerebral ischemia. Neuroprotectin D1 (NPD1), a docosahexaenoic acid (DHA)-derived lipid mediator, promotes cell survival under uncompensated oxidative stress (UOS). Our data demonstrate that NPD1 potently upregulates Iduna expression and provides remarkable cell protection against UOS. Iduna, which was increased by the lipid mediator, requires the presence of the poly(ADP-ribose) (PAR) sites. Moreover, astrocytes and neurons in the penumbra display an enhanced abundance of Iduna, followed by remarkable neurological protection when DHA, a precursor of NPD1, is systemically administered 1 h after 2 h of ischemic stroke. These findings provide a conceptual advancement for survival of neural cells undergoing challenges to homeostasis because a lipid mediator, made 'on demand,' modulates the abundance of a critically important protein for cell survival.

Neuronal cell death involves uncompensated oxidative stress (UOS) as an early event. Parthanatos, a form of cell death, is dependent of poly(ADP-ribose) polymerase-1 (PARP-1).^1, 2, 3, 4^ PARPs catalyze the transfer of ADP-ribose from nicotinamide adenine dinucleotide (NAD) to target proteins and are fundamental for genomic integrity, the cell cycle and gene expression.^5, 6^ Suppression of PARP expression reduces stroke volume.^7, 8^ Iduna (ring finger protein 146 (RNF146) is a PARsylation-directed ring finger E3 ubiquitin ligase that plays a key role in protein quality control, fosters DNA repair and provides protection against parthanatos in cerebral ischemia.^2, 7, 9^ Since it is unknown what key cellular-response signals of neural homeostatic disruptions are linked to Iduna expression, we asked if the lipid mediator neuroprotectin D1 (NPD1) could function in this capacity. NPD1 is derived from the omega-3 docosahexaenoic acid (DHA), which is enriched in the nervous system.^10^ Neuroectoderm-derived post-mitotic retinal pigment epithelial (RPE) cells are protected by NPD1 when exposed to UOS.^11^ Also, during early reperfusion after cerebral ischemia NPD1 synthesis increases in the brain. When NPD1 is administered under these conditions, downregulation of leukocyte infiltration, attenuation of pro-inflammatory signaling and decreased infarct size takes place.^12, 13^ Moreover, systemically administered DHA is used to synthesize NPD1 in the brain after an ischemic stroke. Based on both of these observations in the eye and brain, we decided to explore whether or not NPD1 modulates Iduna expression using RPE cells undergoing UOS^14^ and in an ischemic stroke model.^15^ Our results demonstrate that NPD1 upregulates Iduna expression and provides protection against UOS at the cellular level in a PAR-binding-dependent fashion. Moreover, in ischemic stroke, Iduna expression is downregulated in the penumbra and is associated with neurological deficits. DHA, as a precursor of NPD1, counteracts these deficits by increasing Iduna expression in neurons and astrocytes after stroke onset. Thus our findings establish a causal relationship whereby NPD1 modulates Iduna abundance and cell survival after neural injury/stroke, resulting in neurological protection.

## Results

### NPD1 upregulates Iduna abundance in RPE cells undergoing UOS

**Figure 1:**
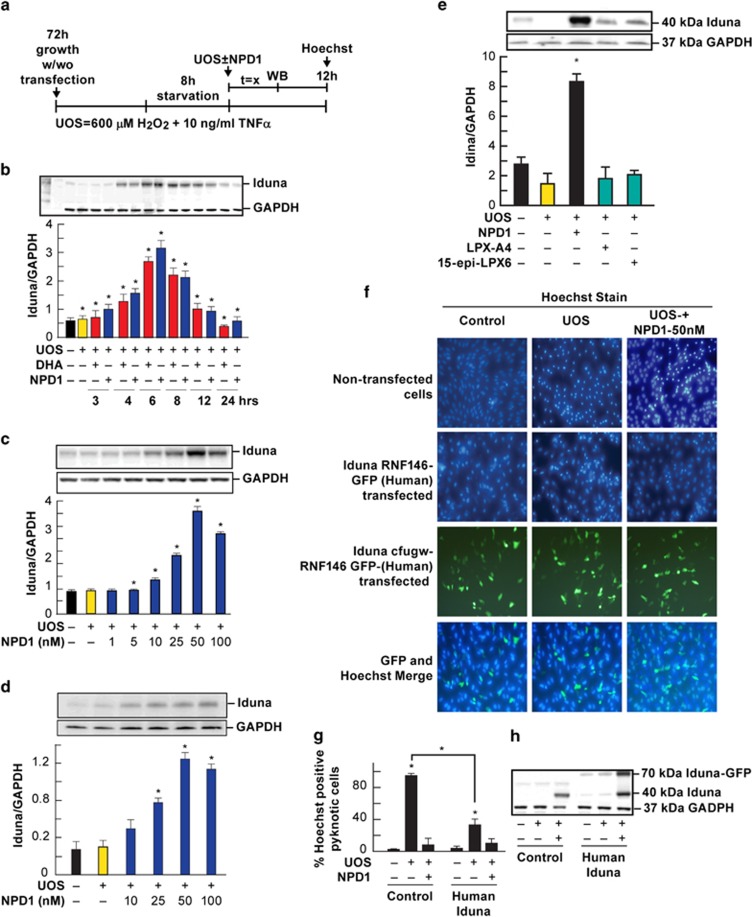
NPD1 upregulates Iduna expression in RPE cells undergoing UOS. (**a**) Experimental design timeline. Cells were transfected and recovered for 72 h. After 8 h of incubation in low serum medium (starvation), UOS was induced with 600 *μ*M H_2_O_2_ plus 10 ng/ml of TNF-*α* in the presence and absence of NPD1. After a variable period (*t*=*x*) that ranged between 3 and 24 h (**b**) or 6 h (**c**–**e** and **h**), cells were harvested for western blot. For Hoechst staining, cells were fixed after 12 h of treatment (**f** and **g**). (**b**) Western blot of the time course of Iduna expression in ARPE-19 cells. (**c** and **d**) NPD1 (1–100 nM)-mediated expression of Iduna in UOS-treated ARPE-19 (**c**) and hRPE cells (**d**). (**e**) Specific expression of Iduna by NPD1, since the lipid mediators lipoxin A4 (LPX-A4) and 15-epi-LPX are inactive in ARPE-19 cells. (**f**) Representative images of Iduna expression by transient transfection of human Iduna construct (Iduna-146-GFP-Hu), (**g**) quantification of Hoechst-positive ARPE-19 cells and (**h**) western blot from transfection with Iduna human constructing ARPE-19 cells undergoing UOS and treated with 50 nM NPD1. Bars represent data averages of three repeats (technical replicas) of three independent experiments (biological replicas). *P<0.05

**Figure 2:**
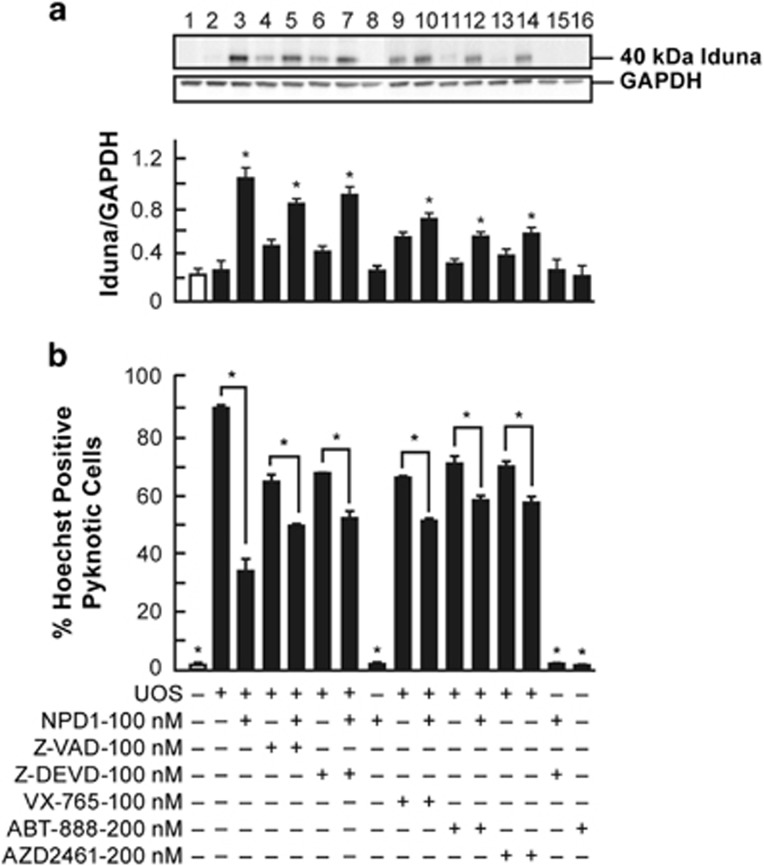
NPD1 enhances survival induced by inhibitors of PARP or caspases. (**a**) Iduna expression was assessed by western blot (upper panel). Densitometry shows expression of Iduna relative to GAPDH. (**b**) Hoechst-positive cell quantification of caspase and PARP inhibitors and their effect on ARPE-19 cells undergoing UOS in the presence and absence of NPD1 (lower panel). Bars represent data averages of three repeats (technical replicas) of three independent experiments (biological replicas). *P<0.05

**Figure 3:**
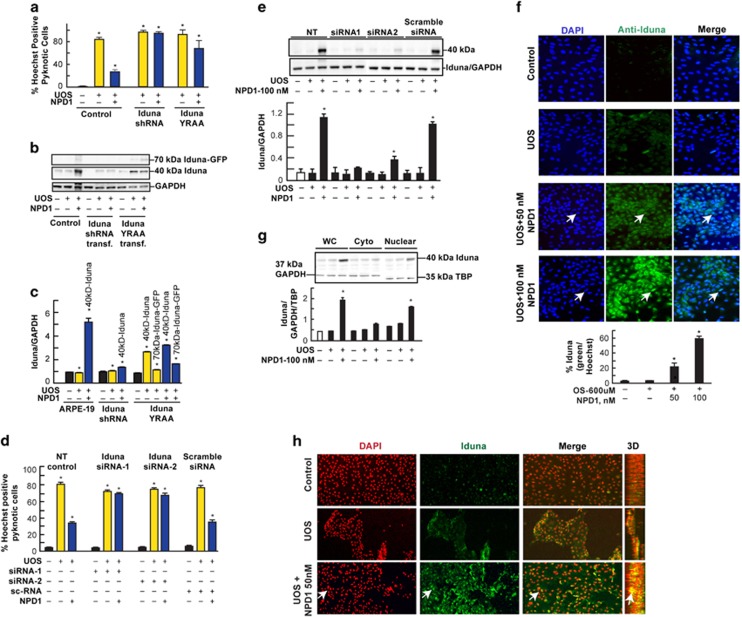
NPD1 mediates expression and translocation of Iduna and PAR dependency. (**a**) Effect of NPD1 on apoptosis in ARPE-19 cells transfected with either shRNA-Iduna or YRAA-Iduna construct. (**b**) Western blot and (**c**) densitometric quantification of Iduna expression by NPD1 in ARPE-19 cells and controls transfected with shRNA-Iduna and YRAA-Iduna construct. (**d** and **e**) Silencing of Iduna using two different siRNAs (siRNA1 and siRNA2) and a control nonspecific targeting siRNA (scramble). (**d**) Hoechst staining of transfected or non-transfected ARPE-19 cells undergoing UOS in the presence or absence of NPD1 and (**e**) Western blot and densitometry of Iduna expression. (**f**) Endogenous expression of Iduna in response to NPD1. Representative images of immunostaining of ARPE-19 cells undergoing UOS in the absence or presence of 50 or 100 nM NPD1 and quantification of Iduna-positive cells. (**f** and **h**) Arrows mark examples of the Iduna-positive nuclei. (**g** and **h**) Nuclear translocation of Iduna by NPD1. (**g**) Western blot analysis and densitometry of whole cell (WC), cytoplasmic (Cyto) and nuclear fractions of ARPE-19 cells undergoing UOS in the presence or absence of 100 nM NPD1. Whole cell and cytoplasmic fractions where standardized using GAPDH and nuclear fraction using TBP. (**h**) Z-stacks flattened 2D images (red=DAPI first column; green=Iduna, second column; merged channels, third column) and 3D representation of ARPE-19 cells immunocytochemistry targeting endogenous Iduna. Bar=200 *μ*m. Arrows indicate co-localization (yellow) of both the DAPI and Iduna signal in the nucleus. Bars represent data averages of three repeats (technical replicas) of three independent experiments (biological replicas). *P<0.05

We used two different types of human RPE cells in separate cultures: (1) a spontaneously transformed cell, ARPE-19, and (2) primary RPE cells. NPD1 enhanced expression of Iduna in both cell types. The effect of NPD1 on Iduna expression peaked at 6 h after the onset of UOS (Figure 1b). A dose-dependent curve showed an increase of Iduna expression starting at 25 nM NPD1 in both cell types (Figures 1c and d). NPD1 and UOS alone did not enhance expression of Iduna (Figures 1b–d, 2a and 3b–h). NPD1 bioactivity was specific since other protective lipid messengers (lipoxin A4 and 15-epi-LPX) were unable to enhance Iduna expression in the RPE cells undergoing UOS (Figure 1e). Altogether these results suggest that NPD1 selectively induces Iduna upregulation, and both NPD1 and UOS are required for Iduna upregulation.

### Iduna overexpression offsets UOS-induced apoptosis in RPE cells

We then tested whether Iduna overexpression would attenuate UOS-stimulated cell death. Overexpression of human Iduna (Iduna-146-GFP-Hu) in RPE cells (Figures 1f and g) attenuated cell death induced by UOS (Figures 1e and f), and the addition of NPD1 (50 nM) to the transfected cells further enhanced inhibition of cell death. Transfection efficiencies of the plasmids were 65 to 70% for the human Iduna construct (Figures 1f, third row). Moreover, the addition of Caspase-3 and Caspase-1 inhibitors, Z-DEVD and VX-765, reduced approximately 25% of the cell death observed in cells undergoing UOS (Figure 2). The addition of 100 nM NPD1 to the caspases inhibitors decreased 43% the level of cell death (Figure 2b) and increased Iduna expression in each case (Figure 2a). The difference in cell death observed between inhibitors and inhibitors plus NPD1 suggest that there is another mechanism of cell death independent of the caspases. Inhibitors of PARP, ABT-888 and AZD2461 showed trends similar to those observed with the caspase inhibitors (Figure 2b). These results suggest that caspase-dependent and PARP-dependent mechanisms are both part of the cell death triggered by UOS, and Iduna expression is involved in the NPD1-mediated protection of RPE cells.

Next we asked whether or not Iduna was involved in the NPD1-mediated survival effects. For this purpose, we used plasmids carrying shRNA-Iduna to silence the protein or YRAA-Iduna. The shRNA was introduced to silence the expression of Iduna, whereas the YRAA-Iduna has a mutation that prevents Iduna from binding with PAR.^2^ Our results show that NPD1 was unable to protect RPE cells against UOS after transfection/expression of either the shRNA-Iduna or YRAA-Iduna constructs (Figure 3a). Cells expressing shRNA-Iduna did not express the protein as expected (Figures 3b and c), and cells transfected with the YRAA-Iduna construct displayed mutant Iduna, which lacks PAR-binding sequences (Figures 3b and c).

### Ablation of PAR-binding sites affects NPD1-mediated Iduna increase

Because PAR regulates protein function through binding at the poly(ADP-ribose) (PAR)-binding sites,^16^ we then asked whether the NPD1-mediated Iduna increase was dependent on PAR binding in RPE cells undergoing UOS. We showed that YRAA mutant Iduna expression in RPE cells reduced the amount of wild-type endogenous Iduna protein in comparison with the non-YRAA-expressing cells undergoing UOS plus NPD1 (Figures 3b and c). In agreement with the latter observation, overexpression of the wild-type Iduna-GFP open reading frame increased the content of endogenous Iduna (Figure 1h). These results suggest that the presence of non-PAR-binding Iduna induce a decrease in the endogenous counterpart and that an interaction between different types of Iduna occurred to modify the overall turnover of the protein (Figures 3b and c). The PARsylation-directed ubiquitination mediated by Iduna is analogous to the phosphorylation-directed ubiquitination catalyzed by the Skp1–Cul1–F-box (SCF) E3 ubiquitin complex.^9, 16^ Therefore, modulation of the PAR-binding activity of Iduna by NPD1 should play a key role in proteasomal protein degradation and cell protection at early stages of neuro-pathophysiology. We found that Iduna expression did not occur in the control cells or in UOS-treated ARPE-19 cells (Figures 3b–h). In contrast, Iduna expression in ARPE-19 cells under UOS is dependent upon the concentration of NPD1 (Figure 3f). Moreover, the nuclear fraction (but not the cytoplasmic fraction) of ARPE-19 cells displayed an increase in the Iduna protein (Figure 3g). In addition, NPD1 induced nuclear Iduna increase (yellow) only in UOS+NPD1-treated cells (Figure 3h, lower row: green in 2D images and yellow in 3D and merged images). Cells that were not treated with NPD1 did not display protein translocation (Figure 3h, upper and middle rows), further supporting the mediator role of NPD1 in this process.

### DHA, as a precursor of NPD1, counteracts ischemic stroke neurological deficits with concomitant increase of Iduna in neurons and astrocytes in the penumbra

**Figure 4:**
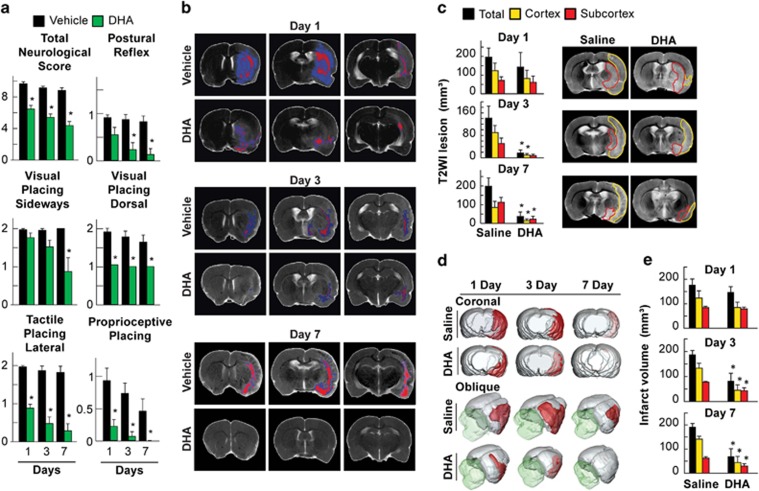
DHA improves neurological scores, protects ischemic penumbra, decreases MRI lesions and diminishes infarct volumes. (**a**) Time course of neurologic assessment (normal score=0, maximal score=12), postural reflex and visual, tactile and proprioceptive forelimb placing reactions (normal score=0, maximal score=2). DHA (*n*=6) or vehicle (*n*=7) were administered 3 h after the onset of MCAo. DHA-treated rats improved all scores on days 1, 3 and 7. (**b**) High-resolution e*x vivo* MRI on days 1, 3 and 7. Core and penumbra were extracted from the entire brain and lesion volumes were determined using MRI. Core (red) and penumbral (blue) tissues were automatically identified in saline- and DHA-treated animals using the computational MRI method Hierarchal Region Splitting for penumbra identification. DHA treatment reduced lesions in the ischemic core and penumbra on days 3 and 7. (**c**) Cortical, subcortical and total lesion volumes, computed from T2WI and representative T2-weighted images (T2WI) from vehicle- and DHA-treated rats on days 1, 3 and 7. DHA treatment reduced T2 values within the lesion on days 3 and 7. T2 hyper-intensities were observed in the cortex and striatum of saline-treated rats, consistent with ongoing edema formation. In contrast, DHA-treated rats had only a small cortical and subcortical lesion on day 7. (**d**) 3D reconstructions of MRI-derived lesion volumes from high-resolution T2WI from vehicle- and DHA-treated rats on days 1, 3 and 7 after MCAo. Vehicle-treated rats showed large cortical and subcortical lesion volumes (red color) that slowly decreased over the course of 7 days. By contrast, lesion volume was dramatically reduced in rats treated with DHA and was localized mostly in the subcortical areas. (**e**) Histopathology on days 1, 3 and 7 after MCAo. Total infarct volume was corrected for brain swelling. DHA treatment dramatically reduced cortical, subcortical and total infarct volumes. Values shown are means±S.D. *P<0.05, *versus* saline group (repeated measures analysis of variance followed by Bonferroni tests)

**Figure 5:**
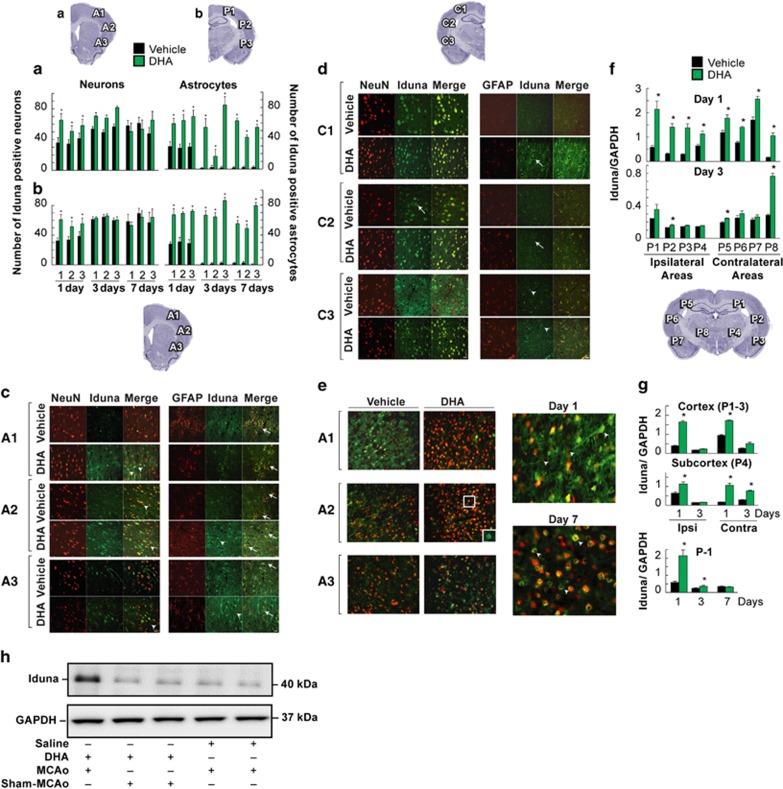
DHA potentiates Iduna expression in neurons and astrocytes. Immunohistochemistry on days 1, 3 and 7 after MCAo. Iduna-positive cells were counted in the cortex at two bregma levels: (**a**) +1.2 mm (A1, A2, A3) and (**b**) −2.8 mm (P1, P2, P3; see diagrams on the top). DHA treatment increased Iduna expression in neurons on day 1 and in astrocytes on days 1, 3 and 7. (**c**) Iduna expression in the neurons and astrocytes from the ischemic cerebral cortexes (see diagram) of vehicle- and DHA-treated rats on day 1. (**d**) Iduna expression in the neurons and astrocytes from the contralateral cerebral cortex (see diagram on the top) from vehicle- and DHA-treated rats on day 1. Iduna expression was mostly located in the cytoplasm of the neurons and also expressed in astrocytes. (**e**) Immunohistochemistry shows a perinuclear Iduna expression compared with vehicle-treated animals (picture inside the white box) on days 1 and 7 after DHA treatment. Iduna expression was observed in the nucleus and projections of the neurons on day 1. Contrary to this, perinuclear localization of Iduna occurred on day 7. (**f**) Western blot: The quantitative data of Iduna expression from ipsi- and contralateral areas on days 1 and 3, and (**g**) cortical and subcortical areas (see diagram) on days 1 and 3 and cortex on days 1, 3 and 7. DHA treatment upregulated the expression of Iduna on day 1 and 3. (**g**) Immunohistochemical detection of Iduna. (**h**) Western blot of ipsilateral penumbra on day 1 from MCAo treated with saline, MCAo treated with DHA and sham-MCAo (no MCA occlusion) treated with DHA groups. Data are mean±S.E.M. *, significantly different from saline (*P*<0.05; repeated measures analysis of variance (ANOVA) followed by Bonferroni tests). Saline *n*=7; DHA *n*=6. Values shown are means±S.D. *P<0.05, *versus* saline group (repeated measures ANOVA followed by Bonferroni tests). Iduna (green); NeuN (red), magnification × 40. Neurons (arrows) and astrocytes (arrowheads)

Having established that NPD1 facilitates Iduna expression at the cellular level, we decided to test whether this mediator also elicits a similar function in ischemic stroke because the neuroprotective bioactivity of this protein has been shown previously under similar conditions. We systemically administered DHA (i.v.) 1 h after the onset of reperfusion following 2 h of middle cerebral artery (MCA) occlusion (MCAo). It has been shown that NPD1 synthesis from systemically administered DHA takes place in the brain under these conditions.^15^ Also, this model of MCAo leads to an improved neurologic score in NPD1-treated rats (Figure 4a).^12^ Our studies show that DHA treatment rescued the ischemic core and penumbra on days 3 and 7 (Figure 4b), decreased edema T2 values (Figures 4c and d) and reduced cortical, subcortical and total lesion volumes (Figure 4e), as shown in the 3D lesion volumes (Figure 4d). Post-ischemic activation of PARP-1 occurs in neurons, astrocytes, microglial cells, endothelia and infiltrating leukocytes (Figure 5). Our data show that systemically administered DHA after MCAo heightened Iduna abundance in neurons on day 1 (Figures 5a and b) and in astrocytes on days 1, 3 and 7 (Figures 5a and b). In addition, sham-MCAo rats treated with DHA showed no increase of Iduna content (Figure 5h). This was ascertained by co-expression with neuron-specific nuclear protein (NeuN) and glial fibrillary acidic protein (GFAP) (Figure 5c). Iduna was augmented 70% in the ipsilesional brain cortex (bregma level +1.2 and −2.8 mm) in DHA-treated animals. Saline-treated animals displayed a 30–40% increase in Iduna abundance one day after MCAo. The presence of Iduna in the contralateral side was cytoplasmatic, whereas in the ipsilesional side Iduna was nuclear (Figure 5d). DHA elicited Iduna localization in the cytoplasm, nucleus and neuronal projections (Figure 5e), and it increased Iduna abundance in the ipsilesional and contralateral cortex and the subcortex on days 1 and 3, compared with vehicle-treated rats (Figure 5f), with no differences observed on day 7 between the DHA- and vehicle-treated animals (Figure 5g).

## Discussion

The present results show that the early neuroinflammatory response mediator NPD1 overcomes cellular damaging events by upregulating the expression of Iduna. We suggest that neural cell homeostasis and function requires induction of critical genes to preserve the integrity of the nervous system. In fact, c-Rel and Birc3 recently were shown to be controlled by NPD1 in a stereoselective-specific manner.^17^ Furthermore, pro- and anti-apoptotic Bcl-2 proteins were shown to be inversely regulated by NPD1 during UOS.^11^ Just as NPD1 also influences the induction of Iduna and the sequestration of the cell death effector PAR, the lipid mediator also targets PP2A dephosphorylation of Ser-62 of Bcl-xL, which, in turn, binds Bax and prevents activation of the mitochondrial events of apoptosis.^18^ These findings provide evidence that clusters of genes might be transcriptionally coordinated by instructions from NPD1 to resist homeostasis disruptions, where Iduna plays a prominent role.

Thus, protective signaling by NPD1-induced Iduna expression in RPE cells are relevant to sight because these cells sustain photoreceptors integrity and their damage participate in early stages of retinal degenerative diseases.^19^ In fact DHA, the precursor of NPD1, is retained by an intercellular loop between these cells^12^ during photoreceptor outer segment renewal initiated by the phagocytosis of their cell tips. Furthermore, genetic deletion of the adiponectin receptor 1 (AdipoR1), independent of the adiponectin cognate ligand, prevents DHA uptake, blunts NPD1 synthesis and results in ensuing photoreceptor degeneration that resembles certain retinal degenerative diseases.^20^ Moreover, it has recently been demonstrated that mutations on the DHA-retention determinant receptor AdipoR1 of photoreceptors leads to certain forms of retinitis pigmentosa^21^ and that a polymorphism of this receptor is involved in age-related macular degeneration in a Finnish population.^22^

Iduna, a PAR-dependent E3 ligase, is involved in protection against neonatal hypoxia–ischemia brain damage. In hypoxia–ischemia encephalopathy, Iduna's downregulation is associated with apoptosis-inducing factor nuclear translocation and neuronal cell death,^23^ and oxidative stressed-DNA damage activates PARP-1.^24^ PARP-1 induces translocation of apoptosis-inducing factor from mitochondria to the nucleus.^25^ Nuclear apoptosis-inducing factor triggers chromatin fragmentation and caspase-independent cell death.^26^ Here we found that inhibitors of PARP alone reduced cell death triggered by oxidative stress in ARPE cells (Figure 2b). When NPD1 was added to the cells treated with PARP inhibitors, a further decrease in cell death was observed with concomitant upregulation of Iduna expression (Figures 2a and b). These results are in agreement with a partial involvement of PARP-dependent cell death. On the other hand, caspase inhibitors did not induce a complete protection alone, and the addition of NPD1 promoted more survival in cells treated with caspase inhibitors. A recent publication shows that in HT22 cells undergoing UOS, Iduna overexpression does not affect mitochondrial dysfunction to cause cytochrome *c* release or activation of caspase-3, but instead inhibits activation of PARP-1 and nuclear apoptosis-inducing factor.^27^ Another set of data, however, shows that the effect of Iduna in MCAo rats is PARP-1-independent.^7^ Our conclusion is that more than one cell death type is involved in ARPE-19 undergoing UOS and that NPD1 protects via Iduna induction, at least in part.

It was proposed that both hemispheres act together synergistically to overcome stroke in young rats.^28^ In agreement with this line of thought, we found that Iduna was overexpressed in response to stroke and DHA treatment in both the ipsilateral and contralateral areas. Moreover, contralateral cortical and subcortical areas showed an increase in Iduna abundance at 3 days *versus* 1 day after MCAo contrary to the ipsilateral counterparts, which involves the penumbra and core of the ischemia–reperfusion lesion. Although Iduna's expression was increased in both hemispheres, subcellular localization differed (Figures 5c, d and f). In the ipsilateral area, Iduna was found in the nucleus, where it may be activated by tankyrase,^9^ and has been shown to regulate the turnover of several proteins involved in DNA repair.^2^ On the other hand, cytoplasmic localization of Iduna was found in the contralateral area. The exact function of cytoplasmic Iduna is still under investigation. It was proposed that binding of Iduna with PAR may happen in the cytoplasm and lead to neuroprotection.^7^ As a PAR-dependent E3 ligase, Iduna displays a variety of substrates, and its final effect depends on the specific context in which the enzyme is activated.

In conclusion, the DHA-derived mediator NPD1 enhances Iduna expression under disruptive neurohomeostasis (e.g., UOS or ischemic stroke after MCAo) and thus serves as a key regulator of cell survival, halting neural cell death. The significance of other DHA-derived mediators and pro-cell survival targets of NPD1 remains to be defined.^14^ Thus, further unraveling of the molecular details of DHA-NPD1-Iduna expression signaling may contribute to possible therapeutic interventions for retinal degenerations and ischemic stroke.

## Materials and methods

### Cell cultures, treatments and subcellular fractionation

ARPE-19 cells were grown and maintained in T-75 mM flasks in DMEM F-12 medium containing 10% FBS and incubated at 37 °C with a constant supply of 5% CO_2_. Similarly, primary human RPE cells were grown in EMEM medium containing 10% FBS and 5% fetal calf serum at 37 °C and 5% CO_2_. Cytoplasmic and nuclear fractions were separated by using the Cell Fractionation Kit of Cell Signaling Technology (Danvers, MA, USA) following the protocol supplied by the manufacturers. In some experiments caspases and PARP inhibitors were used. Caspase-1 inhibitor, VX-765; Caspase-3 inhibitor, Z-DEVD (Selleckchem, Houston, TX, USA); Pan caspase inhibitor, Z-VAD (Tocris Bioscience, Bristol, UK); PARP-1 and -2 inhibitor, ABT-888 and a novel PARP inhibitor, AZD2461 (Selleckchem) were added right before induction of UOS.

### Transient transfection of ARPE-19 cells by Iduna-GFP constructs

ARPE-19 cells (5 × 10^5^) were transfected with 5 *μ*g of human Iduna-GFP constructs (shRNA-Iduna, human YRAA-Iduna) using Fugene-6 according to the manufacturer's protocol (Roach, NJ, USA). In addition, we used Lipofectamine 2000 (Invitrogen, Carlsbad, CA, USA) to transfect cells with siRNAs 1, 2 (Invitrogen-Thermo cat # 4392420 inventory # s37821 and s37822) and scramble (Invitrogen-Thermo cat# AM4611) the predesigned RNA duplexes. Transfected cells were incubated 24 h at 37 °C. They were then serum starved for 8 h at 37 °C. Serum-starved cells were exposed to UOS (600 *μ*M) and treated with NPD1 (50 nM) for 6 h for western blot analysis and 16 h for apoptotic cell death assessment at 37 °C.

### Western blot analysis

ARPE-19/hRPE cells (grown 72 h) in six-well plates were serum starved overnight. UOS was induced by H_2_O_2_ (600*μ*M) plus TNF*α* (10ng/ml), and then cells were challenged with various concentrations (1 nM–2*μ*M) of NPD1 for 3, 4, 6, 8, 10 and 12 h. Treated cells were harvested, cell lysates were made, protein contents were adjusted and western blot analysis was performed using 20–25 *μ*g protein. Proteins were transferred onto PVDF (Invitrogen) paper and the Iduna protein was detected by either anti-Iduna antibody (RNF146) or clone N201/35 containing anti-Iduna (UC Davis/NIH NeiroMab Facility, Davis, CA, USA).

### Immunocytochemistry

Immunocytochemistry was performed on ARPE-19 cells grown on glass slides for 72 h. The cells were serum starved overnight, UOS was induced, and cells treated with 50 and 100 nM NPD1 for 6 h. Then cells were fixed by 4% paraformaldehyde and probed with an Iduna-specific antibody. To detect the NPD1-mediated expression of Iduna in ARPE-19, paraformaldehyde-fixed treated cells were probed with Iduna-specific antibody, followed by Hoechst (2 *μ*M) stain and images were taken by an Axioplan 2 deconvolution microscope and processed with SlideBook 4.2 and 5.0 software (Intelligent Imaging Innovations Inc., Denver, CO, USA).

### Immunohistochemistry

Rats were anesthetized and transcardially perfused with 0.9% saline followed by 4% paraformaldehyde on days 1, 3 and 7 after MCAo. Immunohistochemical procedures were performed on the adjacent sections to identify specific vascular and neuronal elements in the ischemic core and penumbra. Briefly, tissue sections were washed in 1 × PBS and then incubated for 2 h in a blocking solution (5% BSA/0.1 Triton X-100 in 1 × PBS) followed by incubation with the Iduna antibody (1:350; NeuroMab, Davis, CA, USA), NeuN (Chemicon, Temecula, CA, USA) for neurons or GFAP (Cell Signaling, Danvers, MA, USA) to label reactive astrocytes. After incubation, secondary antibodies conjugated with Alexa Flour 488 or 594 were used for 2 h at room temperature. Sections were mounted on superfros Plus slides and coverslipped with ProLong Gold. Images for counting were obtained using an Axioplan 2 deconvolution microscope and processed with SlideBook 4.2 and 5.0 software (Intelligent Imaging Innovations Inc.). We also used a Zeiss LSM-510 Meta laser confocal microscope with × 40 or × 63 oil to take pictures. Thickness for optical slices of all fluorophores was 11.6 *μ*m. Image resolution was set to 3.64 *μ*m/pixel and the z-interval was set to 0.91*μ*m to ensure cubic voxel dimensions. Visualization of fluorescent signal was achieved as follows (excitation; emission; pinhole Ø): AlexaFluor 488 (488 nm; 505 LP; 1.00 Airy) AlexaFluor 594 (594 nm; 543 LP; 1.00 Airy). The number of Iduna-positive cells was counted in the three cortical (ischemic penumbra) and one subcortical (ischemic core) areas (ipsi- and contralateral) at the level of the central lesion (bregma +1.2mm and −1.8; magnification; × 20) using the NIH Image J program. Data were expressed as numbers of positive cells per high-power microscopic. Quantitative assessment was carried out by an investigator blinded to the experimental groups.

### General animal preparation

All experimental protocols were approved by the Institutional Animal Care and Use Committee (IACUC) of the Louisiana State University Health Sciences Center, New Orleans. Male Sprague-Dawley rats (290–320 g; Charles River Lab, Wilmington, MA, USA) were used for *in vivo* studies. For all surgical procedures, animals were fasted and anesthesia was induced with 3.5% isoflurane and 70% nitrous oxide and 30% oxygen. Animals were orotracheally intubated; given atropine for secretions, pancuronium for immobilization; ventilated mechanically on a humidified mixture of 70% nitrous oxide, 1.0–1.5% isoflurane and a balance of oxygen. A femoral artery and vein were catheterized for continuous blood pressure monitoring and periodic blood sampling for arterial gases and pH. PCO_2_ were maintained at 35–40 mm Hg and PO_2_ at 105–120 mm Hg by ventilator adjustments. Rectal temperature was measured with a thermistor and maintained with a heating lamp at 37.0–37.5°C. Cranial (temporalis muscle) temperature also was monitored and regulated with a separate warming lamp at 36.2–36.7°C.

### MCA occlusion

The right MCA was occluded for 2 h by intraluminal filament.^12, 15^ Briefly, the right common carotid artery (CCA) was exposed through an incision in the neck. The CCA was isolated from surrounding nerves. The distal external carotid artery (ECA) and pterygopalatine arteries were tied. A 4-cm of 3-0 monofilament nylon suture coated with poly-lysine was introduced via the proximal ECA into the internal carotid artery and MCA. The correct suture position was confirmed by feeling a certain resistance during filament forwarding or by advancing the suture a defined distance (20–22 mm) from the CCA bifurcation. Then, the animals were allowed to awaken from anesthesia and returned to their cages. The severity of stroke injury was assessed by behavioral examination of each rat at 60 min after the onset of MCAo. Rats that do not demonstrate high-grade contralateral deficit (score, 10–11) were excluded from further study. After 2 h of MCA occlusion, the rats were reanesthetized with the same anesthetic combination. Temperature probes were reinserted, and the intraluminal suture was removed carefully. The neck incision was closed with silk sutures, and the animals were allowed to survive for different times (according to the experimental design) with free access to food and water. Rectal and cranial (temporalis muscle) temperatures, plasma glucose, blood pressure and blood gases showed no significant differences among groups. Sham-operated animals underwent all procedures except for MCAo.

### A composite neurological battery

This battery consisted of two components: (1) a postural reflex test, designed to examine forelimb and upper-body posture in response to tail-suspension and lateral displacement, regarded as being sensitive to both cortical and striatal lesions; (2) an elicited forelimb placing test, which examines sensorimotor integration by assessing placing reactions to visual, tactile and proprioceptive stimuli. The total neurologic score was graded on a scale from 0 (normal) to 12 (maximal deficit), as we previously described.^12, 15^

### Treatments

DHA (5 mg/kg; Cayman, Ann Arbor, MI, USA; *n*=6) or vehicle (0.9% saline; *n*=7) was administered intravenously into the femoral vein at 3 h after the onset of MCAo. Rats were randomly allocated to experimental groups and treatments.

### Magnetic resonance imaging acquisition

High-resolution *ex vivo* magnetoc resonance imaging (MRI) was performed on 4% paraformaldehyde-fixed brains at each time point (days 1, 3 and 7) using an 11.7T Bruker Advance 8.9 cm horizontal bore instrument equipped with an 89 mm (ID) receiver coil (Bruker Biospin, Billerica, MA, USA). T2-weighted images (T2WI), diffusion weighted images (DWI), 3D volumes and apparent diffusion coefficient (ADC) maps were collected as we previously described.^29^

### MRI image analysis: lesion, core and penumbra volumes

T2 and ADC maps were computed from T2WI and DWI, respectively. We used hierarchical region splitting (HRS) to automatically identify core and penumbra volumes (total lesion= core+penumbra) from T2 relaxation and water mobility (ADC), as we have published previously.^29^ Our penumbral tissue determination by HRS was confirmed by use of PWI/DWI subtractions at each brain level, as we have done previously.^29^ The penumbra will be defined as the difference between the PWI and abnormal ADC (diffusion−perfusion mismatch) (2 STD elevation or reduction compared to normal tissues).

### Statistical analysis

Data are presented as mean values±S.D. Repeated measure analysis of variance followed by Bonferroni procedures were used for multiple comparisons. Two-tailed Student's *t*-tests were used for two-group comparisons. Differences at *P*<0.05 were considered statistically significant. Behavioral testing, data acquisition and analysis were performed in a blinded manner.
